# Tissue Inhibitor of Metalloproteinase-2 Polymorphisms and Risk for HIV-Associated Neurocognitive Disorder

**DOI:** 10.1155/2019/8278095

**Published:** 2019-05-28

**Authors:** HariOm Singh, Sushma Jadhav, Dharmesh Samani, Sumitra Nain

**Affiliations:** ^1^Department of Molecular Biology, National AIDS Research Institute, Pune 411026, India; ^2^Department of Pharmacy, University of Banasthali, Banasthali Vidyapith, Jaipur 302001, India

## Abstract

The imbalance between MMPs and TIMPs is associated with the HIV dissemination tissue damage pathology neurodegenerative disorders, including HAND. Genetic variations in the *TIMP* gene may modulate the neurocognitive disorder in HIV patients. Hence, we evaluated the genetic variants of *TIMP*-2 (*-*418G/C, 303G/A) gene with the risk of HAND. Genotyping of *TIMP*-2 polymorphism was performed in 50 patients with HAND, 100 no HAND, and 154 healthy controls by PCR-RFLP. *TIMP-2* -418GC and 303AA genotypes represented a predominant risk for HAND severity (OR = 1.55, *P* = 0.30; OR = 4.58, *P* = 0.24). The variant -418CC genotype, -418A allele, had exhibited a significant risk for the acquisition of HAND (OR = 12.55, *P* = 0.026; OR = 2.66, *P* = 0.004). *TIMP-2* 303GA, 303AA genotype, and 303A allele evinced a higher risk for HAND severity (OR = 1.82, *P* = 0.14; OR = 1.70, *P* = 0.63; and OR = 1.68, *P* = 0.12). In HIV patients, *TIMP-2* -418CC genotype and -418C allele significantly occurred in comparison to healthy controls (OR = 10.10, *P* = 0.006; OR = 2.02, *P* = 0.009). In the intermediate and early HIV disease stage, *TIMP-2* -418CC genotype was significantly increased compared with healthy controls (11.1% vs. 1.3%, OR = 14.63, *P* = 0.01; 16.9% vs. 1.3%, OR = 14.51, *P* = 0.002). In patients with HAND among tobacco and alcohol users, *TIMP-2* -418CC genotype displayed a risk for HAND severity (OR = 3.96, *P* = 0.26; OR = 4.83, *P* = 0.19). On multivariate logistic regression, *TIMP-2* 303AA genotype, advanced stage, and gender had a risk for HAND severity (OR = 28.98, *P* = 0.02; OR = 2.35, *P* = 0.070; and OR = 2.36, *P* = 0.04). In conclusion, *TIMP-2* -418G/C polymorphism independently, along with alcohol and tobacco, may have an impact on the acquisition of HAND and its severity. *TIMP-2* 303G/A polymorphism bare a risk for HAND severity.

## 1. Introduction

Human immunodeficiency virus (HIV) directly invades the brain in a short period following the infection. The central nervous system (CNS) effects by replicating in macrophages and microglia and enhancing the inflammatory and neurotoxic responses in the host [1]. HIV-associated neurocognitive disorder (HAND) is a complication that occurs in HIV-infected patients. HAND is a neurocognitive impairment that has an asymptomatic neurocognitive impairment (ANI), mild neurocognitive disorder (MND), and HIV-associated dementia (HAD) [2]. Studies from India have reported a very high prevalence of HAND (32.50% and 35%) [3]. According to a study, the incidence of development of HIV-associated dementia (HAD) was nearly about 20-30% in a precombined antiretroviral therapy (cART) era and reduced to 10% in the post-cART era. Thus, the occurrence of HAD was found notably reduced in the post-cART era [4]. The occurrence of HAD is estimated to be nearly 6% in India [5, 6]. HIV subtypes (clade), dose-dependent drug reactions, coinfections, and extracellular matrix (ECM) proteases are important in the modulation of HAND pathogenesis [7–9]. Extracellular matrix, a notable proportion of the CNS, contributes to alterations of ECM in the brain and may lead to mild cognitive impairment (MCI) [10]. An imbalance ratio of the matrix metalloproteinases (MMPs)/tissue inhibitors of matrix metalloproteinases (TIMPs) is associated with degradation of the ECM. The pathogenesis of inflammation-related diseases is associated with the degradation of the ECM [11]. The disparity between MMPs and TIMPs is mainly concerned with neurodegenerative diseases including Alzheimer's disease, ischemia, HAD, and other coinfection in HIV such as hepatitis C [12–14]. Due to the neurotoxic properties, MMPs and endogenous tissue inhibitors of MMPs (TIMPs) are widely studied to elucidate the pathogenesis of HAND [15]. Irregular expression of MMPs and TIMPs disturbs the blood-brain barrier (BBB) and allows the neurotoxic substance to penetrate into nerve cells leading to cell death [16, 17]. In HAND patients, the plasma level of MMP-2/TIMP-2 was significantly higher as compared to the patients without HAND [15]. Collectively, active ECM is degraded by MMPs, which is tightly regulated by its inhibitor TIMPs. TIMP is a family of antiproteinases, is composed of four members (TIMP-1, 2, 3, and 4), and inhibits the activity of MMPs. Specifically, the MMP-2 and TIMP-2 play a significant role in maintaining the integrity in healthy tissues and regulate cell growth and apoptosis [18].

A secretary protein, TIMP-2, is located at 17q25, which inhibits the proteolytic activity of matrix metalloproteinase 2 (MMP-2) [19]. The genetic variations of *MMP-2* and *TIMP-2* are linked to low transcriptional activity and with the risk of several diseases [20–23]. *TIMP-*2 (-418G/C, 303G/A) polymorphisms are located in the promoter region and known to have an influence on the transcriptional activity and occurrence of the tumor [20, 24, 25]. Genetic variation of *TIMP-2* is also associated with the risk of the malignant tumor and cancer [23, 26]. *TIMP*-2 (-418G/C, 303G/A) polymorphisms and haplotype CGC were associated with the risk of gastric cancer and abdominal aortic aneurysm (AAA) [27, 28]. The GT (*TIMP-2 -*418^∗^G/303^∗^T) haplotype was associated with an increased risk of prostate cancer (OR = 1.78; 95% CI: 1.18-2.69, *P* = 0.006, Pc = 0.024) [29]. *TIMP-2* -418GC genotype showed a protective effect against prostate cancer (32.6% vs. 14.8%, *P* = 0.037, OR = 0.346) [30]. Although no association of *TIMP-2* gene polymorphisms with the increased risk of prostate cancer, cervical cancer, myocardial infarction (MI) or coronary artery disease (CAD), and relapsing-remitting multiple sclerosis (RRMS) was reported [29, 31–33]. However, genetic variation of *TIMP-2* (-418G/C, 303G/A) gene in patients with and without HAND is still unknown. Hence, the present study is projected to evaluate the genetic variants of *TIMP-2* (-418G/C and 303G/A) gene with the risk of HAND from Western India.

## 2. Materials and Methods

### 2.1. Subjects

The number of patients with HAND was 50, which were enrolled from December 2013 to November 2015 with the confirmation of International HIV-associated Dementia Score (IHDS) <9.5 and documented evidence for HIV positive. The number of patients without HAND was 100, which were also recruited and were showing IHDS score >9.5 indicating without HAND status from the outdoor patient clinics of the National AIDS Research Institute, Pune. Patients having concurrent untreated opportunistic infections and febrile illness currently or in the past 15 days were excluded from HAND and without HAND groups. Simultaneously, one hundred fifty-four age and ethnicity-matched healthy controls with the negative status of hepatitis B and C, tuberculosis, and HIV serum negative from SD Bioline ELISA test were recruited from the same clinics. The questionnaire, personal interviews, and review of case records were used to collect the patient's clinical data. The guidelines of IHDS 2005 (grant 2008) were used for dementia scaling by the clinician. Estimation of the CD4 cell count was done by FACS. CD4 counts <200 cells/mm^3^, 200-350 cells/mm^3^, and >350 cells/mm^3^ were considered as advanced, intermediate, and early stage of HIV infection, respectively. The predesigned questionnaire was used to record the tobacco and alcohol usage. The institutional ethics committee (NARI/EC-2011/06 dated 13th October 2011) approved the study, a written and signed informed consent (ICF version 1.0 dated 18 April 2011) was taken from all participants.

### 2.2. DNA Extraction

Two ml peripheral blood sample was collected and stored at -700°C prior to DNA extraction. Genomic DNA extraction was done from the peripheral blood leukocyte pellet using the Blood Genomic DNA Miniprep Kit of AxyPrep (Axygen Biosciences) according to the protocol given by the manufacturer.

### 2.3. Genotyping

Polymerase chain reaction-restriction fragment length polymorphism (PCR-RFLP) was used to genotype the *TIMP*-2 (-418G/C and 303G/A) polymorphisms. Primers for the amplification of *TIMP*-2 -418G/C and 303G/A were taken as described [28, 34]. PCR was performed in a total volume of 25 *μ*l with 10 pmol of each primer, genomic DNA (100-150 ng), 2.5 mM deoxynucleotide triphosphates, PCR buffer containing 100 mM Tris-HCl, pH 8.6, 50 mM KCl, 1.5 mM MgCl2, and 1.5 units of Taq polymerase (Bangalore Genies, India). The reaction conditions for *TIMP*-2 -418G/C were initial denaturation at 94°C for 3 min, followed by 35 cycles of denaturation at 94°C for 30 sec, annealing at 62°C for 1 min, extension at 72°C for 1 min, and a final extension at 72°C for 7 min. The reaction conditions for *TIMP*-2 303G/A were initial denaturation at 94°C for 3 min, followed by 35 cycles of denaturation at 94°C for 30 sec, annealing at 64°C for 1 min, extension at 72°C for 1 min, and a final extension at 72°C for 7 min. All reactions were carried out in Thermal Cycler Model 2720 (Applied Biosystems, USA). PCR products and molecular weight markers were visualized in 2% agarose gel for confirmation of amplification. The expected amplicon size for *TIMP-2*-418G/C and 303G/A gene is 304 bp and 119 bp, respectively. The PCR product of *TIMP*-2 -418G/C and 303G/A was digested using restriction enzyme *AvaI* and *TspRI* (MBI Fermentas Inc.)*. TIMP*-2 -418G/C genotyping was done in 15% polyacrylamide gel using molecular weight markers and visualized after staining with ethidium bromide. *TIMP*-2 -418G/C genotyping was done based on the sequences and location of SNP, as assigned as follows: 230 bp, 51 bp, and 23 bp for -418GG; 253 bp, 230 bp, 51 bp, and 23 bp for -418GC; and 253 and 51 bp for -418CC genotype and 103 bp and 16 bp for 303GG; 119 bp, 103 bp, and 16 bp for 303GA; and 119 bp for 303AA. Other laboratory personnel did the regenotyping in 20% of samples from both patients and controls to rule out the technical discrepancy in genotyping. The genotyping error was assessed in 10% of samples by sequencing.

### 2.4. Statistical Analysis

The mean age variable was expressed as mean ± standard deviation (SD). The deviation from the Hardy-Weinberg equilibrium in controls was calculated by *χ*^2^ goodness-of -fit test. We used the *χ*^2^ statistic (Fisher's exact test for cell size <5) to compare the genotype frequency between HIV patients with HAND vs. without HAND, HIV patients vs. healthy controls. Unconditional binary logistic regression analysis was used to derive the odds ratio (OR) and 95% confidence interval (CI). SPSS software version 17.0 was used for statistical analysis, the two-sided value was used for the tests of statistical significance, and *P* value less than ≤0.05 was considered for significance. SNPStats online analysis tool was used to compare haplotype frequency among the respective groups [35]. Linkage disequilibrium (LD) was estimated between both the loci by calculating the relative LD value (*D*′) as *D*′ = Dij/*D*max [36]. The Dij values were compared between patients with HAND and without HAND, HIV patients, and healthy controls.

## 3. Results

In the present study, we have included 50 patients with HAND, 100 patients without HAND, and 154 healthy individuals. The mean age (years ± SD) of HIV patients with HAND, HIV patients without HAND, and healthy individuals was 40.34 ± 3.41, 39.45 ± 7.34, and 38.23 ± 5.7, respectively. The characteristics of HIV patients with HAND, HIV patients without HAND, and healthy individuals are shown in Table 1.

### 3.1. *TIMP-2* (*-*418G/C and 303G/A) Polymorphisms and Patients with HAND

Frequency distribution of *TIMP-2* (*-*418G/C and 303G/A) polymorphism in patients with HAND and no HAND is shown in Table 2. The predominance of *TIMP-2* -418GC, 303GA, and 303AA genotypes and 303A allele was found to be higher in HAND patients compared with no HAND (30.0% vs. 20.0%, OR = 1.55, 95% CI: 0.68-3.51, *P* = 0.30; 34.0% vs. 28.0%, OR = 1.36, 95% CI: 0.64-2.89, *P* = 0.42; 4.0% vs.1.0%, OR = 4.58, 95% CI: 0.37-56.83, *P* = 0.24; and 21.0% vs.15.0%, OR = 1.57, 95% CI: 0.83-2.30, *P* = 0.17, respectively). Frequency of *TIMP-2* -418CC genotype and -418C allele was distributed nearly alike between patients with HAND and without HAND (8.0% vs.13.0%, OR = 0.69, 95% CI: 0.20-2.32, *P* = 0.55; 23.0% vs.23.0%, OR = 0.99, 95% CI: 0.55-1.78, *P* = 0.99, respectively). In patients with HAND, the prevalence of *TIMP-2* -418GC and -418CC genotypes and -418C allele was observed to be higher when compared with healthy controls (30.0% vs. 22.1%, OR = 2.19, 95% CI: 0.93-5.15, *P* = 0.072; 8.0% vs.1.3%, OR = 12.55, 95% CI: 1.36-115.90, *P* = 0.026; and 23.0% vs. 12.3%, OR = 2.66, 95% CI: 1.36-5.23, *P* = 0.004, respectively). The incidence of *TIMP-2* 303GA and 303AA genotypes and 303A allele was seemed to be higher in patients with HAND as compared to healthy controls (34.0% vs. 29.2%, OR = 1.82, 95% CI: 0.82-4.053, *P* = 0.14; 4.0% vs. 1.9%, OR = 1.70, 95% CI: 0.20-14.64, *P* = 0.63; and 21.0% vs. 16.6%, OR = 1.68, 95% CI: 0.88-3.23, *P* = 0.12, respectively)

### 3.2. *TIMP-2* (*-*418G/C and 303G/A) Polymorphism and HIV Patients

The frequency distribution of *TIMP-2* (*-*418G/C and 303G/A) genotype/allele in HIV patients and healthy individuals is presented in Table 3. *TIMP-2 -*418CC genotype and -418C allele were significantly overrepresented in HIV patients as compared to healthy controls (13.0% vs. 1.3%, OR = 10.10, 95% CI: 1.97-51.68, *P* = 0.006; 23.0% vs. 12.3%, OR = 2.02, 95% CI: 1.20-3.41, *P* = 0.009, respectively). Occurrence of *TIMP-*2 303GA and 303GG genotype and 303A allele was not different between HIV patients and healthy controls (28.0% vs. 29.2%, OR = 1.01, 95% CI: 0.54-1.89, *P* = 0.97; 1.0% vs. 1.9%, OR = 0.34, 95% CI: 0.025-4.66, *P* = 0.42; and 15.0% vs. 16.6%, OR = 0.84, 95% CI: 0.49-1.46, *P* = 0.54, respectively).

### 3.3. Gene-Gene Interaction

The distribution of haplotype frequency of *TIMP-2* (*-*418G/C and 303G/A) polymorphisms in respective groups is shown in Table 4. While comparing patients with HAND vs. without HAND, HAND patients vs. healthy controls, and HIV patients vs. healthy individuals, the linkage disequilibrium (LD) *D*′ between both the genes (*D*′ = 0.068, 0.049, and 0.014) was analyzed by the SNPStats online analysis tool. No significant differences were found in the comparison of LD values (Dij) between *TIMP-2* polymorphisms (*P* = 0.32, 0.84, and 0.75). It was anticipated that there could be an additive effect of these variations with the acquisition of HAND and its severity. In gene-gene interaction analysis, haplotype GG (*TIMP-2 -*418^∗^G and 303^∗^G) was considered as a reference. Occurrence of haplotypes GA and CA **(***-*418^∗^G/303^∗^A, -418^∗^C/303^∗^A) was higher in HAND patients while comparing with no HAND (0.15 vs.0.10, OR = 1.70, 95% CI: 0.77-3.74, *P* = 0.19; 0.05 vs. 0.04, OR = 1.34, 95% CI: 0.32-5.63, *P* = 0.69, respectively). In patients with HAND, haplotype CG (*-*418^∗^C/303^∗^G) occurred significantly higher in comparison with healthy controls (0.17 vs.0.10, OR = 2.55, 95% CI: 1.05-6.18, *P* = 0.04). On comparing between HAND patients and healthy controls, haplotypes GA and CA (*-*418^∗^G/303^∗^A, *-*418^∗^C/303^∗^A) occurred more frequent in HAND cases (0.16 vs. 0.15, OR = 1.57, 95% CI: 0.69-3.59, *P* = 0.28; 0.05 vs.0.01, OR = 4.75, 95% CI: 0.58-38.60, *P* = 0.15, respectively). In HIV patients, haplotypes CG and CA (*-*418^∗^C/303^∗^A, -418^∗^C/303^∗^A) were distributed predominantly higher in comparison to healthy controls **(**0.18 vs. 0.10, OR = 1.70, 95% CI: 0.94-3.08, *P* = 0.08; 0.04 vs. 0.01, OR = 1.99, 95% CI: 0.45-8.77, *P* = 0.37, respectively).

### 3.4. *TIMP-2* (*-*418G/C and 303G/A) Polymorphism and HIV Disease Stages

The incidence of *TIMP-2* -418 CC genotype was found to be significantly higher in early and intermediate HIV disease stages compared with healthy controls (16.9% vs. 1.3%, OR = 14.51, 95% CI: 2.66-79.09, *P* = 0.002; 11.1% vs. 1.3%, OR = 14.63, 95% CI: 1.91-112.25, *P* = 0.01, respectively). The prevalence of *TIMP-2* 303GA genotype was almost alike between individuals with early HIV disease stage and healthy controls (33.9% vs. 29.2%, OR = 1.39, 95% CI: 0.69-2.83, *P* = 0.36) (Table 5).

### 3.5. Interaction between Gene and Environment

In gene-environment analysis, we evaluated the risk for HAND in the presence of *TIMP-2* polymorphisms and alcohol and tobacco usage. In tobacco using patients with HAND, the prevalence of *TIMP-2 -*418CC genotype was much frequent than nonusers (15.4% vs. 5.4%, OR = 3.96, 95% CI: 0.37-42.62, *P* = 0.26) (Table 6). Similarly, the distribution of *TIMP-2* -418CC, 303GA, and 303AA genotypes was much more often in alcohol-consuming patients with HAND compared to no HAND (18.2% vs. 5.1%, OR = 4.83, 95% CI: 0.47-50.04, *P* = 0.19; 36.4% vs. 33.3%, OR = 1.91, 95% CI: 0.38-9.57, *P* = 0.43; and 9.1% vs. 2.6%, OR = 6.58, 95% CI: 0.31-137.56, *P* = 0.23, respectively). In alcohol-consuming HIV patients, *TIMP-2* -418CC and 303GA genotypes occurred much than nonusers (24.1% vs. 10.7%, OR = 2.03, 95% CI: 0.52-7.95, *P* = 0.31; 31.0% vs. 25.0%, OR = 1.72, 95% CI: 0.55-5.34, *P* = 0.35) (Table 7).

### 3.6. Risk Factors of HAND: Multivariate Logistic Regression Analysis

We have looked the relationship of *TIMP-2 -*418 G/C and 303 G/A polymorphisms, age, sex, tobacco, alcohol, and HIV disease stages with HAND was done by multivariate logistic regression analysis. *TIMP-2* 303AA genotype, advanced disease stage (CD4 <200), intermediate disease stage (CD4 201-350), and sex appeared as independent risk factor for HAND severity (OR = 28.98, *P* = 0.02; OR = 2.35, *P* = 0.07; *P* ≤ 0.001, OR = 6.65; and *P* = 0.04, OR = 2.36, respectively). Tobacco users, *TIMP-2* 303GA genotype, have shown a risk for HAND severity (OR = 3.65, *P* = 0.42; OR = 1.83, *P* = 0.16, respectively). *TIMP-2 -*418G/C polymorphism, age, and alcohol consumption have not shown a risk for HAND (Table 8).

## 4. Discussion

The degradation of ECM by MMPs is tightly regulated by tissue inhibitors of MMPs. The imbalance between MMPs and TIMPs affects the stability of BBB and also alters the activation of monocytes [15]. A higher ration of MMP-2/TIMP-2 plasma concentrations was reported in HAND patients [15]. Genetic variations in *TIMP-2* gene may interrupt the balance, associated with the diseases susceptibility [37]. *TIMP-2* -418G/C and 303G/A polymorphisms alter the expression profile and spatial conformation [24]. In the present study, the genotype distribution of *TIMP-2* -418G/C gene in healthy individuals was almost similar with studies carried out by Zhang et al. and Srivastava et al. [28, 29] but differed from studies described by Mikolajczyk-Stecyna et al., Yaykasli et al., Aksoy et al., Alp et al., and Srivastava et al. [27, 30–33]. Similarly, the genotype distribution of *TIMP-2* 303G/A gene in healthy controls was comparable with the study described by Srivastava et al. [29] and incomparable with a study done by Zhang et al. [28].

In this study, while comparing between patients with and without HAND vs. healthy controls, we found that *TIMP-2* -418CC genotype and -418C allele were associated with the acquisition of HAND and its severity (OR = 10.10, *P* = 0.006; OR = 2.66, *P* = 0.004; and OR = 12.55, *P* = 0.03). It is hypothesized that *TIMP-2* -418G/C polymorphism may have a role in the acquisition of both HAND and its severity. On comparing between patients with and without HAND, we have observed that *TIMP-2* 303AA genotype has shown a risk for HAND severity (OR = 4.58, *P* = 0.24). The prevalence of the *TIMP-2* -418CC genotype and C allele was associated with abdominal aortic aneurysm (AAA) patients [27]. *TIMP-2* 303G/A and -418G/C polymorphisms were associated with gastric cancer patient [28]. *TIMP-2* -418GC genotype was associated with the reduced risk of prostate cancer (*P* = 0.037, OR = 0.346) [30].

Genetic variants of *TIMP-2* -418G/C gene were not associated with patients of relapsing-remitting multiple sclerosis (RRMS) [31]. *TIMP-2* -418G/C and 303C/T polymorphisms were associated neither with the risk of developing prostate cancer nor with the risk of cervical cancer [29, 33].

In the present study, we have also analyzed the haplotypes among the groups. While comparing HAND patients and healthy controls, we found that haplotype CG was associated with HAND patients (OR = 2.55, *P* = 0.04). Similarly, when we compared between HIV patients and healthy controls, we found haplotype CG was likely to be associated with the risk for the development of HAND (OR = 1.70, *P* = 0.08). This suggests that haplotype CG might have a role in the acquisition of HAND and its severity. The frequency of haplotype CGC (*MMP-2*-1306C/T, *TIMP-2* -418G/C and 303G/A) was apparently higher in patients with gastric cancer than the control group (*P*<0.05) [28]. Haplotype results demonstrated that *TIMP-2* (-418G/303T) was associated with a 1.8-fold increased risk of prostate cancer [29].

Present study was designed as case-control in which current CD4 cell count was taken as a substitute for disease progression. Since we do not know the exact time for the acquisition of HIV, the outcomes may be irritating by the time interval of HIV infection. In subgroup analysis, *TIMP-2* -418CC genotype was associated with individuals of intermediate HIV disease stage compared to healthy controls (OR = 14.63, *P* = 0.01). Individuals having intermediate HIV disease stage with *TIMP-2* -418CC genotype may facilitate the risk for the advancement of HIV disease (OR = 14.63, *P* = 0.01).

We have also attempted to analyze the gene-environment interaction to determine the etiology of disease [38, 39]. To look at the interaction between gene and environment, we have performed a case-only analysis. We had not done a case-control analysis because the cases have to match with the controls in the population, then it may lead untrue interactions [40]. In an individual with HIV infection, consuming alcohol had a harmful effect on the CD4 cell count [41]. In our study, in patients with HAND using tobacco and alcohol, *TIMP-2 -*418CC genotype has shown a risk for HAND severity (OR = 3.96, *P* = 0.26 and OR = 4.83, *P* = 0.19). In alcohol-consuming patients with HAND, *TIMP-2* 303GA and 303AA genotypes revealed a risk for HAND severity (OR = 1.91, *P* = 0.43 and OR = 6.58, *P* = 0.23). In HIV patients consuming alcohol, *TIMP-2 -*418CC and 303GA genotypes have presented the risk for the acquisition of HAND (OR = 2.03, *P* = 0.31 and OR = 1.72, *P* = 0.35). *MMP*-2 and *TIMP-2* polymorphisms in the presence of tobacco usage were not associated with the risk of prostate cancer [29]. There was no significant association between *TIMP-2* -418G/C gene polymorphism and cervical cancer risk due to tobacco usage (OR = 1.70, *P* = 0.139) [33]. In multivariate logistic regression, we compared between patients with and without HAND, we found that *TIMP-2* 303AA genotype, advanced disease stage (CD4 <200), intermediate disease stage (CD4 201-350), and sex were associated with severity of HAND (OR = 28.98, *P* = 0.02, OR = 2.35, *P* = 0.07, OR = 6.65, *P* ≤ 0.001, OR = 2.36, *P* = 0.04). It is hypothesized that HAND patients with *TIMP-2* 303G/A polymorphism, advanced, and intermediate stages are more susceptible to severity of HAND.

## 5. Conclusion

*TIMP-2*-418G/C polymorphism and its haplotype could be linked with the acquisition of HAND, its severity, and advancement of the disease. Also, this polymorphism can facilitate the risk of HAND severity in tobacco- and alcohol-consuming patients with HAND. *TIMP-2* 303G/A polymorphism has also shown a risk for the HAND severity. The role of MMPs and TIMPs is diverse in the pathogenesis of the neurological disorders, and genetic polymorphisms have been associated with HIV diseases. Hence, the present study should be validated. Further study on *TIMP* and *MMP* polymorphisms in patients with HAND should be carried out in other population with larger samples.

## Figures and Tables

**Table 1:** Characteristics of HIV patients with HAND, without HAND, and healthy controls.

Subjects	HIV patients HAND on ART (years ± SD)	HIV patients on ART (years ± SD)	Healthy controls (years ± SD)
Number of participants	50	100	154
Mean age and standard deviation	39.20 + 6.55	38.28 + 7.62	30.32 + 8.27
Males	23	65	113
Females	27	35	41
Ethnicity	Western India	Western India	Western India
*Alcohol habit*			
Users	11	29	0
Nonusers	39	56	0
*Tobacco habits*			
Users	13	19	0
Non-users	37	66	0
*CD4 status*			
Advance stage (0-201)	29	14	NA
Intermediate stage (201-350)	20	27	NA
Early stage (351 above)	1	59	NA

HAND = HIV-associated neurological disorders; ART = antiretroviral therapy; SD = standard deviation; NA = not applicable.

**Table 2:** Frequency distribution of *TIMP-2* (-418G/C and 303G/A) polymorphism in patients with vs. without HAND and patients with HAND vs. healthy controls.

Genotypes *TIMP-2* -418G/C	Patients with HAND (*N* = 50) (%)	Patients without HAND (*N* = 100) (%)	*P* value	OR (95% CI)
GG	31 (62.0%)	67 (67.0%)	1	Reference
GC	15 (30.0%)	20 (20.0%)	0.30	1.55 (0.68-3.51)
CC	4 (8.0%)	13 (13.0%)	0.55	0.69 (0.20-2.32)
Alleles *TIMP-2* -418G/C	Patients with HAND (*N* = 100) (%)	Patients without HAND (*N* = 200) (%)	*P* value	OR (95% CI)
G	77 (77.0%)	154 (77.0%)	1	Reference
C	23 (23.0%)	46 (23.0%)	0.99	0.99 (0.55-1.78)
Genotypes *TIMP-2* 303G/A	Patients with HAND (*N* = 50) (%)	Patients without HAND (*N* = 100) (%)	*P* value	OR (95% CI)
GG	31 (62.0%)	71 (71.0%)	1	Reference
GA	17 (34.0%)	28 (28.0%)	0.42	1.36 (0.64-2.89)
AA	2 (4.0%)	1 (1.0%)	0.24	4.58 (0.37-56.83)
Alleles *TIMP-2* 303G/A	Patients with HAND (*N* = 100) (%)	Patients without HAND (*N* = 200) (%)	*P* value	OR (95% CI)
G	79 (79.0%)	170 (85.0%)	1	Reference
A	21 (21.0%)	30 (15.0%)	0.17	1.57 (0.83-2.30)
Genotypes *TIMP-2* -418G/C	Patients with HAND (*N* = 50) (%)	Healthy controls (*N* = 154) (%)	*P* value	OR (95% CI)
GG	31 (62.0%)	118 (76.6%)	1	Reference
GC	15 (30.0%)	34 (22.1%)	0.07	2.19 (0.93-5.15)
CC	4 (8.0%)	2 (1.3%)	**0.03**	**12.55 (1.36-115.90)**
Alleles *TIMP-2*-418G/C	Patients with HAND (*N* = 100) (%)	Healthy controls (*N* = 308) (%)	*P* value	OR (95% CI)
G	77 (77.0%)	270 (87.7%)	1	Reference
C	23 (23.0%)	38 (12.3%)	**0.004**	**2.66 (1.36-5.23)**
Genotypes *TIMP-2* 303G/A	Patients with HAND (*N* = 50) (%)	Healthy controls (*N* = 154) (%)	*P* value	OR ( 95% CI)^a^
**GG**	31 (62.0%)	106 (68.8%)	1	Reference
**GA**	17 (34.0%)	45 (29.2%)	0.14	1.82 (0.82-4.053)
**AA**	2 (4.0%)	3 (1.9%)	0.63	1.70 (0.20-14.64)
Alleles *TIMP-2* 303G/A	Patients with HAND (*N* = 100) (%)	Healthy controls (*N* = 308) (%)	*P* value	OR (95% CI)
G	79 (79.0%)	257 (83.4%)	1	Reference
A	21 (21.0%)	51 (16.6%)	0.12	1.68 (0.88-3.23)

*N* = total number of subjects, (%) = frequency of genotypes/alleles, age-adjusted OR (odds ratio) and 95% CI (confidence intervals) were derived from logistic regression models comparing the homozygous wild-type genotype/allele (GG genotype and G allele for *TIMP-2* -418G/C and 303G/A were taken as reference) with other genotypes/alleles. Significant *P* values (*P* < 0.05) and related OR (95% CI) have been shown in bold.

**Table 3:** Frequency distribution of *TIMP-2* (-418G/C and 303G/A) polymorphisms in HIV patients without HAND and healthy controls.

Genotypes *TIMP-2* -418G/C	Patients without HAND (*N* = 100) (%)	Healthy controls (*N* = 154) (%)	*P* value	OR (95% CI)
GG	67 (67.0%)	118 (76.6%)	1	Reference
GC	20 (20.0%)	34 (22.1%)	0.92	1.04 (0.52-2.08)
CC	13 (13.0%)	2 (1.3%)	**0.006**	**10.10 (1.97-51.68)**
Alleles *TIMP-2* -418G/C	Patients without HAND (*N* = 200) (%)	Healthy controls (*N* = 308) (%)	*P* value	OR (95% CI)
G	154 (77.0%)	270 (87.7%)	1	Reference
C	46 (23.0%)	38 (12.3%)	**0.009**	**2.02 (1.20-3.41)**
Genotypes *TIMP-2* 303G/A	Patients without HAND (N=100) (%)	Healthy controls (*N* = 154) (%)	*P* value	OR (95% CI)
**GG**	71 (71.0%)	106 (68.8%)	1	Reference
**GA**	28 (28.0%)	45 (29.2%)	0.97	1.01 (0.54-1.89)
**AA**	1 (1.0%)	3 (1.9%)	0.42	0.34 (0.025-4.66)
Alleles *TIMP-2* 303G/A	Patients without HAND (*N* = 200)	Healthy controls (*N* = 308) (%)	*P* value	OR (95% CI)
G	170 (85.0%)	257 (83.4%)	1	Reference
A	30 (15.0%)	51 (16.6%)	0.54	0.84 (0.49-1.46)

*N* = total number of subjects, (%) = frequency of genotypes/alleles; age-adjusted OR (odds ratios) and 95% CI (confidence intervals) were derived from logistic regression models comparing the homozygous wild-type genotype/allele (GG genotype and G allele for *TIMP-2* -418G/C and 303G/A were taken as reference) with other genotypes/alleles. Significant *P* values (*P* < 0.05) and related OR (95% CI) have been shown in bold.

**Table 4:** Frequency distribution of haplotypes of *TIMP-2* (-418G/C and 303G/A) polymorphisms among patients with HAND, without HAND, and healthy controls.

Haplotypes *TIMP-2* (*-*418G/C and 303G/A)	Patients with HAND (*N* = 100)	Patients without HAND (*N* = 200)	*P* value	OR (95% CI)
**GG**	0.61	0.66	1	Reference
**CG**	0.17	0.18	0.94	1.03 (0.52 - 2.02)
**GA**	0.15	0.10	0.19	1.70 (0.77 - 3.74)
**CA**	0.05	0.04	0.69	1.34 (0.32 - 5.63)
Haplotypes *TIMP-2* (*-*418G/C and 303G/A)	Patients with HAND (*N* = 100)	Healthy controls (*N* = 308)	*P* value	OR ( 95% CI)
**GG**	0.61	0.72	1	Reference
**CG**	0.17	0.10	**0.04**	**2.55 (1.05 - 6.18)**
**GA**	0.16	0.15	0.28	1.57 (0.69 - 3.59)
**CA**	0.05	0.01	0.15	4.75 (0.58 - 38.60)
Haplotypes *TIMP-2* (-418G/C and 303G/A)	HIV patients (*N* = 200)	Healthy controls (*N* = 308)	*P* value	OR ( 95% CI)
**GG**	0.66	0.72	1	Reference
**CG**	0.18	0.10	0.08	1.70 (0.94 - 3.08)
**GA**	0.10	0.15	0.61	0.84 (0.43 - 1.64)
**CA**	0.04	0.01	0.37	1.99 (0.45 - 8.77)

*N* = total number of alleles, (%) = frequency of haplotypes; age-adjusted odds ratios and 95% CIs were derived from logistic regression models comparing the haplotype GG with other haplotypes. Significant *P* values (*P* < 0.05) and related OR (95% CI) have been shown in bold.

**Table 5:** Frequency distribution of *TIMP-2* (*-*418G/C and 303G/A) polymorphisms in HIV disease stages of HIV patients (CD4 <200, 201-350, and >350) and healthy controls.

Genotypes *TIMP-2* -418G/C	Healthy controls (*N* = 154) (%)	Early HIV disease stage		Intermediate HIV disease stage		Advanced HIV disease stage	
		*N* = 59 (%)	OR (*P*)	*N* = 27 (%)	OR (*P*)	*N* = 14 (%)	OR (*P*)
GG	118 (76.6%)	35 (59.3%)	Reference	20 (74.1%)	Reference	12 (85.7%)	Reference
GC	34 (22.1%)	14 (23.7%)	1.35 (0.46)	4 (14.8%)	0.70 (0.57)	2 (14.3%)	0.65 (0.60)
CC	2 (1.3%)	10 (16.9%)	**14.51 (0.002)**	3 (11.1%)	**14.63 (0.01)**	0 (0.0%)	NS
Genotypes *TIMP-2* 303G/A	Healthy controls (*N* = 154) (%)	Early HIV disease stage		Intermediate HIV disease stage		Advanced HIV disease stage	
		*N* = 59 (%)	OR (*P*)	*N* = 27 (%)	OR (*P*)	*N* = 14 (%)	OR (*P*)
GG	106 (68.8%)	38 (68.8%)	Reference	21 (77.8%)	Reference	12 (85.7%)	Reference
GA	45 (29.2%)	20 (33.9%)	1.39 (0.36)	6 (22.2%)	0.86 (0.77)	2 (14.3%)	0.50 (0.39)
AA	3 (1.9%)	1 (1.7%)	0.67 (0.76)	0 (0.0%)	NS	0 (0.0%)	NS

*N* = number of subjects, (%) = frequency of subjects; odds ratios and 95% CIs were derived from logistic regression models comparing the homozygous wild-type genotype (GG genotype for *TIMP-2* -418G/C and 303G/A was taken as reference) with other genotypes. Significant *P* values (*P* < 0.05) and related OR have been shown in bold.

**Table 6:** Frequency distribution of *TIMP-2* (-418G/C and 303G/A) genotypes in tobacco using HIV patients with and without HAND.

HIV patients with HAND				
Genotypes *TIMP-2* -418G/C	Tobacco users	Nonusers	*P* value	OR (95% CI)
	*N* = 13 (%)	*N* = 37 (%)		
GG	7 (53.8%)	24 (64.9%)	1	(Reference)
GC	4 (30.8%)	11 (29.7%)	0.76	1.27 (0.27-6.03)
CC	2 (15.4%)	2 (5.4%)	0.26	3.96 (0.37-42.62)
Genotypes *TIMP-2* 303G/A	Tobacco users	Nonusers	*P* value	OR (95% CI)
	*N* = 13 (%)	*N* = 37 (%)		
GG	9 (69.2%)	22 (59.5%)	1	(Reference)
GA	4 (30.8%)	13 (35.1%)	0.79	0.81 (0.18-3.65)
AA	0 (0.0%)	2 (5.4%)	NS	—
HIV patients without HAND				
Genotypes *TIMP-2-* 418G/C	Tobacco users	Nonusers	*P* value	OR (95% CI)
	*N* = 19 (%)	*N* = 66 (%)		
GG	13 (68.4%)	43 (65.2%)	1	(Reference)
GC	3 (15.8%)	13 (19.7%)	0.96	1.03 (0.22-4.80)
CC	3 (15.8%)	10 (15.2%)	0.72	0.76 (0.17-3.44)
Genotypes *TIMP-2* 303G/A	Tobacco users	Nonusers	*P* value	OR (95% CI)
	*N* = 19 (%)	*N* = 66 (%)		
GG	15 (78.9%)	47 (71.2%)	1	(Reference)
GA	4 (21.1%)	19 (28.8%)	0.56	0.68 (0.19-2.50)
AA	0 (0.0%)	0 (0.0%)	NS	NS

*N* = number of subjects, (%) **=** frequency of subjects; odds ratios and 95% CIs were derived from logistic regression models comparing the homozygous wild-type genotype (GG genotype for *TIMP-2* -418G/C and 303G/A was taken as reference) with other genotypes. Tobacco status in 15 HIV-infected individuals was unknown, therefore excluded from the analysis.

**Table 7:** Frequency distribution of *TIMP-2* (-418G/C and 303G/A) genotypes in alcohol using HIV patients with and without HAND.

HIV patients with HAND				
Genotypes *TIMP-2 -*418G/C	Alcohol users	Nonusers	*P* value	OR (95% CI)
	*N* = 11 (%)	*N* = 39 (%)		
GG	6 (54.5%)	25 (69.1%)	1	(Reference)
GC	3 (27.3%)	12 (30.8%)	0.83	1.20 (0.23-6.31)
CC	2 (18.2%)	2 (5.1%)	0.19	4.83 (0.47-50.04)
Genotypes *TIMP-2* 303G/A	Alcohol users	Nonusers	*P* value	OR (95% CI)
	*N* = 11 (%)	*N* = 39 (%)		
GG	6 (54.5%)	25 (64.1%)	1	(Reference)
GA	4 (36.4%)	13 (33.3%)	0.43	1.91 (0.38-9.57)
AA	1 (9.1%)	1 (2.6%)	0.23	6.58 (0.31-137.56)
HIV patients without HAND				
Genotypes *TIMP-2 -*418G/C	Alcohol users	Nonusers	*P* value	OR (95% CI)
	*N* = 29 (%)	*N* = 56 (%)		
GG	20 (69.0%)	36 (64.3%)	1	(Reference)
GC	2 (6.9%)	14 (25.0%)	0.16	0.30 (0.057-1.61)
CC	7 (24.1%)	6 (10.7%)	0.31	2.03 (0.52-7.95)
Genotypes	Alcohol users	Non-users	*P* value	OR (95% CI)
	*N* = 29 (%)	*N* = 56 (%)		
*TIMP-*2 303G/A				
GG	20 (69.0%)	42 (75.0%)	1	(Reference)
GA	9 (31.0%)	14 (25.0%)	0.35	1.72 (0.55-5.34)
AA	0 (0.0%)	0 (0.0%)	NS	NS

*N* = number of subjects, (%) = frequency of subjects; odds ratios and 95% CIs were derived from logistic regression models comparing the homozygous wild-type genotype (GG genotype for *TIMP-2* -418G/C and 303G/A was taken as reference) with other genotypes. Alcohol status in 15 HIV-infected individuals was unknown, therefore excluded from the analysis.

**Table 8:** Multivariate analysis between patients with and without HAND.

Variables	B	S.E.	df	*P* value	OR (95% CI)
-418GG			2	0.67	
-418GC	0.357	0.460	1	0.43	1.42 (0.58-3.52)
-418CC	-0.189	0.66	1	0.77	0.82 (0.22-3.05)
303GG			2	0.04	
303GA	0.60	0.43	1	0.16	1.83 (0.78-4.29)
303AA	3.36	1.48	1	**0.02**	**28.98 (1.59-527-23)**
			2	0.001	
Intermediate disease stage (CD4 201-350)	1.895	0.525	1	**<0.001**	**6.65 (2.37-18.63)**
Advanced disease stage (CD4 <200)	0.857	0.473	1	0.07	2.35 (2.35-5.95)
Age	0.048	0.030	1	0.10	1.05 (0.99-1.11)
Sex	0.861	0.429	1	**0.04**	**2.36 (1.02-5.47)**
Tobacco user	1.296	0.637	1	0.42	3.65 (1.04-12.73)
Alcohol user	-1.038	.634	1	0.10	0.35 (0.10-1.22)

*TIMP-2* -418G/C and 303G/A polymorphism; age 18-50 years, sex, tobacco user, alcohol user, baseline CD4. Significant values (<0.05) represented and related OR (95% CI) have been shown in bold.

## Data Availability

The data used to support the findings of this study are included within the article.

## Abbreviations

MMP: Matrix metalloproteinase
TIMP: Tissue inhibitor of matrix metalloproteinase
HIV: Human immunodeficiency virus
HAND: HIV-associated neurocognitive disorder
HAD: HIV-associated dementia
cART: Combined antiretroviral therapy
ECM: Extracellular matrix
CNS: Central nervous system
MI: Myocardial infarction
CAD: Coronary artery disease
RRMS: Relapsing-remitting multiple sclerosis
IHDS: International HIV-associated dementia score
ELISA: Enzyme-linked immunosorbent assay.
